# Catalytic asymmetric reactions of isocyanides for constructing non-central chirality

**DOI:** 10.3762/bjoc.21.129

**Published:** 2025-08-19

**Authors:** Jia-Yu Liao

**Affiliations:** 1 College of Pharmaceutical Sciences, Zhejiang University, Hangzhou, 310058, China

**Keywords:** axial chirality, helical chirality, inherent chirality, isocyanide, planar chirality

## Abstract

Beyond the conventional carbon-centered chirality, catalytic asymmetric transformations of isocyanides have recently emerged as a powerful strategy for the efficient synthesis of structurally diverse scaffolds featuring axial, planar, helical, and inherent chirality. Herein, we summarize the exciting achievements in this rapidly evolving field. These elegant examples have been organized and presented based on the reaction type as well as the resulting chirality form. Additionally, we provide a perspective on the current limitations and future opportunities, aiming to inspire further advances in this area.

## Introduction

**Figure 1:**
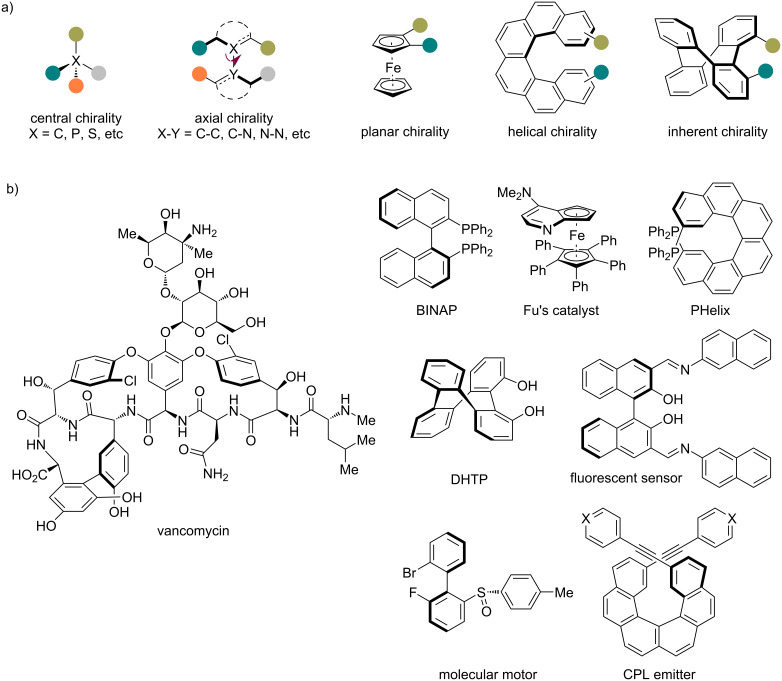
a) Common types of chirality. b) Representative functional molecules bearing non-central chirality.

Chirality represents a fundamental property of molecules and manifests in diverse forms (Figure 1a). While central chirality based on stereogenic centers (e.g., C, P, S, etc.) is the most conventional type, non-central chirality, such as axial [1–4], planar [5–7], helical [8–10], and inherent chirality [11–12], has gained increasing attention due to its broad applications in various fields, including but not limited to drug discovery, asymmetric catalysis, and materials science (Figure 1b). Consequently, the development of efficient and stereoselective methods for assembling such scaffolds with respect to structural diversity has become a hot topic in synthetic organic chemistry.

Isocyanides (also termed isonitriles) are a class of highly versatile building blocks in organic synthesis, participating in a wide range of transformations including multicomponent reactions (e.g., the well-known Passerini and Ugi reactions) [13–15], insertion reactions [16–18], cycloaddition reactions (e.g., [4 + 1], [3 + 2]) [19–20], and others [21–23]. Particularly, isocyanides have been widely exploited toward the preparation of centrally chiral structures through transition-metal-catalyzed or organocatalytic asymmetric reactions [24–26]. Beyond these great developments, recent efforts have successfully expanded the utility of isocyanides to access structurally diverse non-central chiral frameworks, further expanding their synthetic potential. The following sections highlight these fruitful achievements, organized by both the reaction type and the chirality type of resulting products.

## Perspective

### Isocyanide-based transformations

#### Palladium-catalyzed isocyanide insertion reactions

**Scheme 1:**
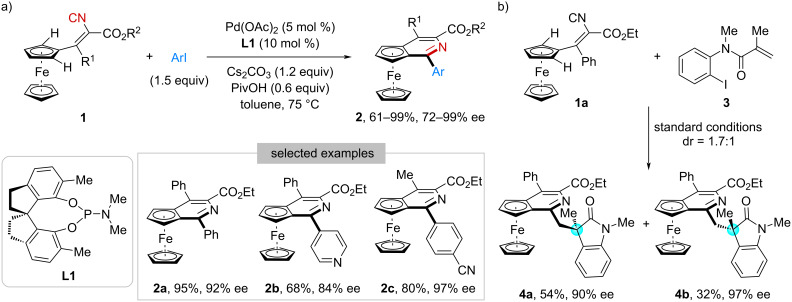
Construction of planar chirality.

In 2018, Luo, Zhu, and co-workers developed a palladium-catalyzed enantioselective reaction between ferrocene-derived vinyl isocyanides **1** and aryl iodides (Scheme 1a) [27]. This transformation proceeded via two key steps, isocyanide insertion and desymmetric C(sp^2^)–H bond activation. By using phosphoramidite **L1** as the chiral ligand, planar chiral pyridoferrocenes **2** were obtained in 61–99% yield with 72–99% ee. In addition, this catalytic system could be applied to synthesize more complex structures. As shown in Scheme 1b, when *N*-(2-iodophenyl)methacrylamide **3** and **1a** were employed as starting materials, compound **4** bearing nonadjacent planar and central chirality was obtained in good yield and enantioselectivity (**4a**, 54%, 90% ee; **4b**, 32%, 97% ee). However, the diastereoselectivity is modest (1.7:1), likely resulting from insufficient chiral induction during the indolinone-forming step.

**Scheme 2:**
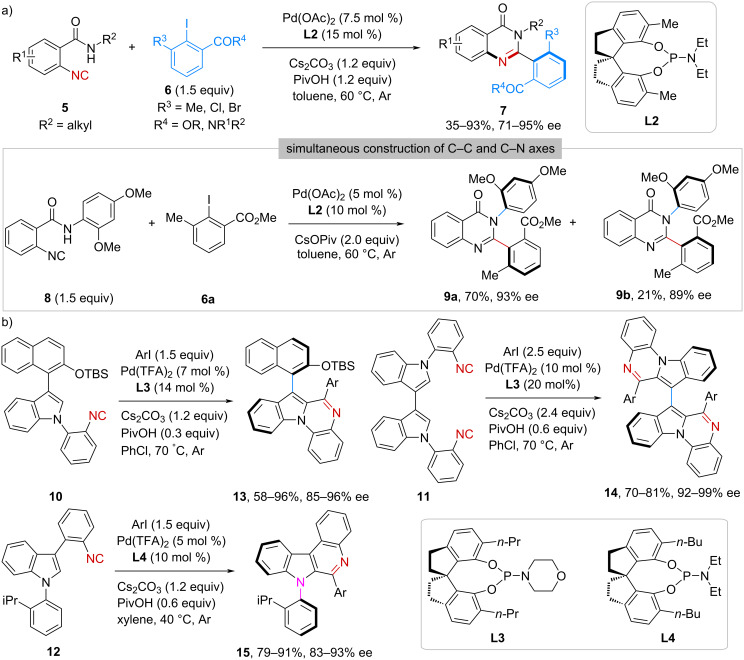
Construction of axial chirality.

Moving forward, this strategy was applied in the construction of axial chirality by the same group. In 2021, they reported a Pd(OAc)_2_/**L2**-catalyzed imidoylative cycloamidation of *N*-alkyl-2-isocyanobenzamides **5** with 2,6-disubstituted aryl iodides **6** (Scheme 2a) [28]. Through a coupling–cyclization reaction sequence, axially chiral 2-arylquinazolinones **7** were synthesized in 35–93% yield with 71–95% ee. Interestingly, by using *N*-(2,4-dimethoxyphenyl)-2-isocyanobenzamide (**8**) and aryl iodide **6a** as the reactants, diastereomeric products **9a** and **9b**, each containing two distinct stereogenic axes (C–C and C–N), were obtained in 93% and 89% ee, respectively.

Very recently, Luo and co-workers implemented an efficient palladium-catalyzed atroposelective C(sp^2^)–H imidoylative cyclization of functionalized phenyl isocyanides, guided by DFT calculations (Scheme 2b) [29]. Three types of isocyanides (**10**–**12**) were evaluated in reactions with aryl iodides, affording indole-fused N-heteroaryl scaffolds **13**–**15**, featuring either a C–C or C–N stereogenic axis, in moderate-to-high yields with high enantioselectivities.

**Scheme 3:**
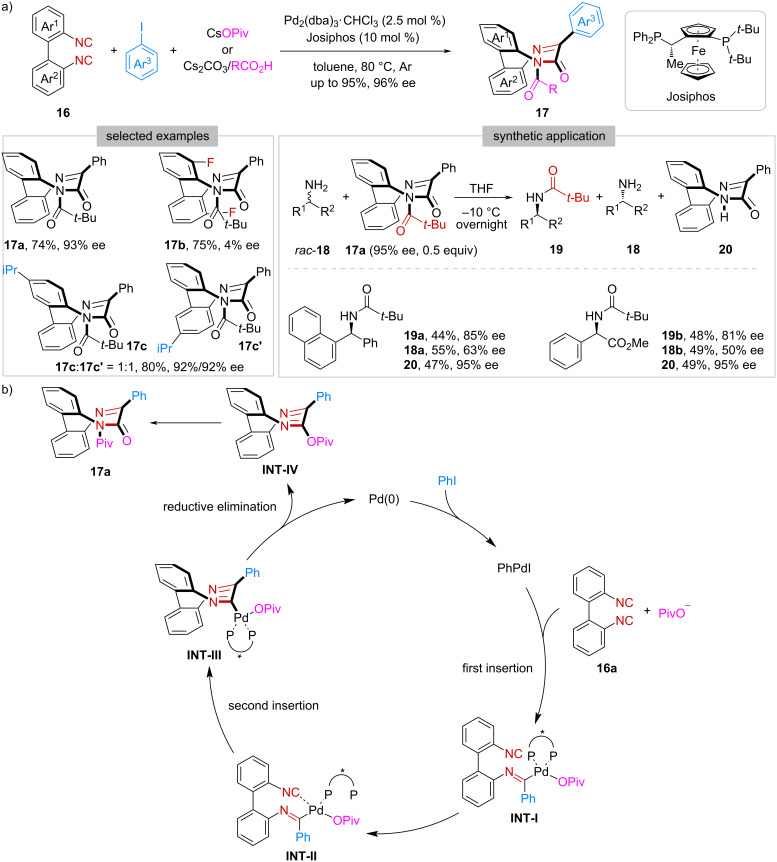
Construction of inherent chirality.

Beyond planar and axial chirality, the same group developed a three-component coupling reaction of 2,2′-diisocyano-1,1′-biphenyls **16**, aryl iodides, and carboxylates (Scheme 3a) [30]. Under chiral palladium catalysis, unique inherently chiral saddle-shaped bridged biaryls **17** were formed through a reaction sequence involving double isocyanide insertion, reductive elimination, and acyl transfer. To be noted, the introduction of a substituent at the *ortho*-position of the isocyanide group in **16** caused a significant drop in the enantioselectivity (e.g., **17b**). Besides, when unsymmetrical diisocyanide was used, the initial isocyanide insertion was found to be non-regioselective, delivering a 1:1 mixture of regioisomers (**17c** and **17c’**). Intriguingly, **17a** could act as a chiral acylating reagent, applying in the kinetic resolution of racemic primary amines *rac*-**18**. Additionally, after the reaction, the resulting deacylated compound **20** could be recovered in almost quantitative yield without any erosion of the enantiopurity. A possible reaction mechanism for this Pd-catalyzed three-component reaction was proposed (Scheme 3b). As shown, the reaction started with the oxidative addition of phenyl iodide to Pd(0) to generate the phenyl Pd(II) species. After that, coordination and migratory insertion of the first isocyanide group of **16a** to Pd(II) delivered **INT-I**. Then, coordination and migratory insertion of the second isocyanide group occurred to give **INT-III**, which underwent reductive elimination to afford **INT-IV**. Finally, migration of the Piv group from O to N gave the product **17a**.

**Scheme 4:**
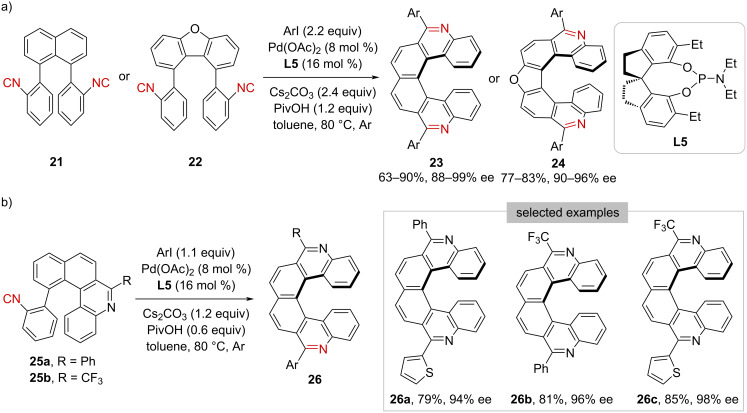
Construction of helical chirality.

Moreover, the Pd-catalyzed isocyanide insertion approach has been successfully extended to the generation of helical chirality [31]. As shown in Scheme 4a, phenyl diisocyanides **21** or **22** underwent double C(sp^2^)–H imidoylative cyclization with aryl iodides, furnishing symmetrical pyrido[6]helicenes **23** or furan-incorporating pyrido[7]helicenes **24** with stable helical chirality, respectively. Furthermore, pre-cyclized monoisocyanides, such as **25a** and **25b**, were identified as another class of suitable substrates under the standard conditions (Scheme 4b). On one hand, such findings demonstrated that the second cyclization determines enantioselectivity; on the other hand, it provided a practical way to the preparation of unsymmetrical pyrido[6]helicenes **26**.

### Isocyanide-based multicomponent reactions

**Scheme 5:**
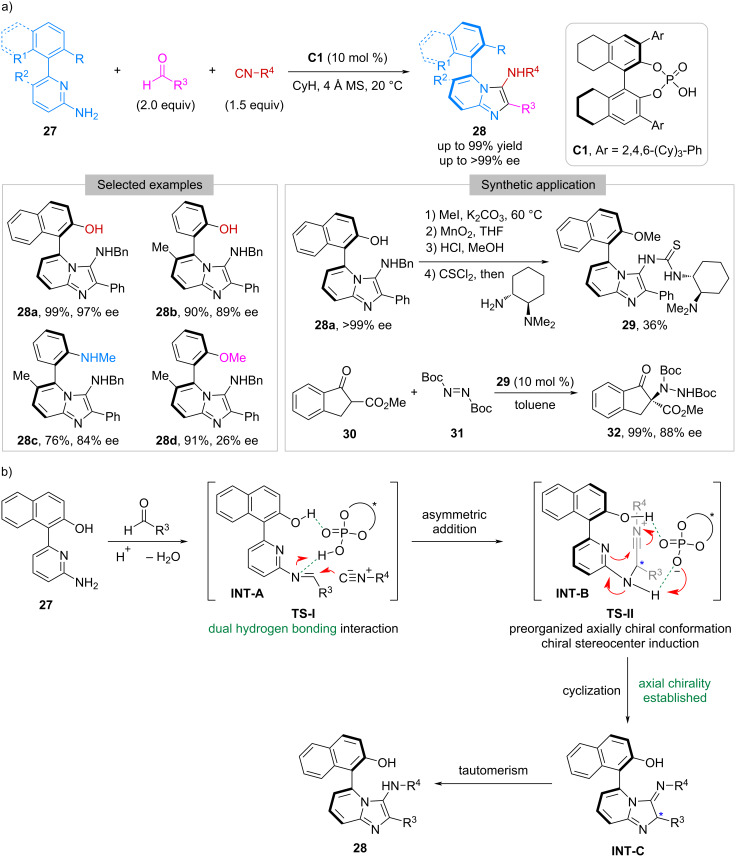
CPA-catalyzed enantioselective Groebke–Blackburn–Bienaymé reaction.

Except for Pd-catalyzed isocyanide insertion reactions, organocatalytic isocyanide-based multicomponent reactions have been explored for the synthesis of axially chiral compounds. In 2024, Yang and co-workers reported a catalytic asymmetric version of the Groebke–Blackburn–Bienaymé reaction [32–34] involving 6-aryl-2-aminopyridines **27**, aldehydes, and isocyanides (Scheme 5a) [35]. By employing chiral phosphoric acid (CPA) **C1** as the catalyst, this reaction worked well to afford axially chiral imidazo[1,2-*a*]pyridines **28** in high-to-excellent yields (up to 99%) and enantioselectivities (up to >99% ee). It is worth noting that the presence of a hydrogen bonding donor in **27** is crucial for achieving high enantioselectivity. As shown, while replacing the OH with NHMe led to a slight decrease of ee (**28c** versus **28b**), the protection of the OH with methyl caused a severe drop (**28d** versus **28b**). The application of the resulting products in developing chiral organocatalysts was investigated as well. For instance, **28a** was converted to a thiourea-tertiary amine **29** through a four-step procedure in an overall 36% yield. This compound was then utilized as the catalyst in the electrophilic amination reaction between β-ketoester **30** and di-*tert*-butyl azodicarboxylate (**31**), and the corresponding product **32** was obtained in 99% yield with 88% ee. A plausible reaction mechanism was proposed for this CPA-catalyzed enantioselective Groebke–Blackburn–Bienaymé reaction. As illustrated in Scheme 5b, the imine condensation between **27** and the aldehyde afforded **INT-A**, which was activated by the CPA catalyst through hydrogen bonding interaction. The nucleophilic addition of isocyanide to **Int-A** produced **INT-B** bearing a stereogenic center. Subsequently, **INT-B** underwent intramolecular cyclization to generate axially chiral **INT-C**, which, after imine-enamine tautomerization, led to the formation of final product **28**.

### α-Acidic isocyanide-based transformations

#### De novo arene formation

**Scheme 6:**
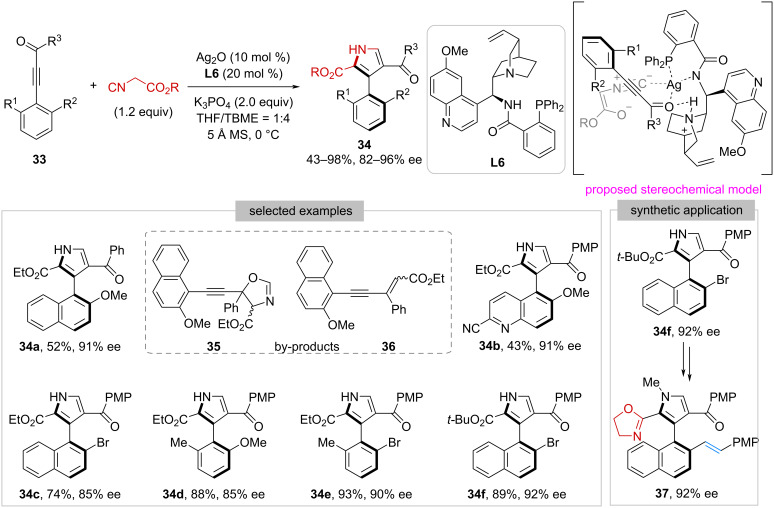
Construction of axially chiral 3-arylpyrroles via de novo pyrrole formation.

In 2019, Zhu and co-workers developed the first example of catalytic enantioselective Yamamoto–de Meijere pyrrole synthesis [36–37] between alkynyl ketones **33** and isocyanoacetates (Scheme 6) [38]. The success of this study not only adds a new entry to the de novo arene formation strategy [39–40] but also initiates the application of isocyanoacetates in constructing axial chirality. With Ag_2_O and quinine-derived amino-phosphine ligand **L6** as the chiral catalyst, atropisomeric 3-arylpyrroles **34** were generated in 43–98% yield with 82–96% ee. Notably, two by-products **35** and **36** were observed during the reaction, resulting from the aldol reaction of isocyanoacetates with the ketone moiety in **33**. The authors have also demonstrated that **34f** could be used as the starting material to prepare the axially chiral olefin-oxazole **37**, which might be a potentially useful ligand in asymmetric catalysis. A possible stereochemical model was proposed as well, involving synergistic activation of both the alkynyl ketone and isocyanoacetate by the chiral catalyst.

#### Central-to-axial chirality transfer

**Scheme 7:**
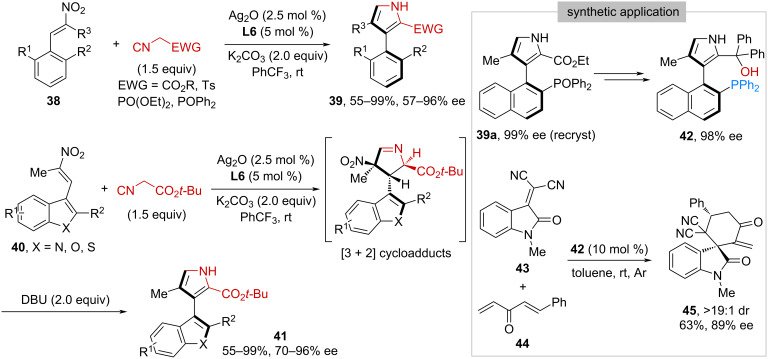
Synthesis of atropoisomeric 3-arylpyrroles via central-to-axial chirality transfer.

In parallel with Zhu’s work, Du, Chen, and co-workers reported an alternative way for the preparation of axially chiral 3-arylpyrroles [41] through a catalytic asymmetric Barton–Zard reaction [42] via central-to-axial chirality transfer strategy [43–44]. Under a similar silver-based catalytic system, nitroolefins **38** bearing a β-*ortho*-substituted aryl group reacted smoothly with α-acidic isocyanides to give the corresponding products **39** in 55–99% yield and 57–96% ee (Scheme 7). In addition, nitroolefins **40** possessing a β-five-membered heteroaryl ring were proven to be suitable substrates to react with *tert*-butyl isocyanoacetate, affording 3-heteroarylpyrroles **41** in 55–99% yield with 70–96% ee. In these cases, additional 2.0 equivalents of DBU were required to facilitate the conversion from the [3 + 2] cycloadducts to the final products. Moreover, an axially chiral tertiary alcohol-phosphine **42** was prepared from **39a** through a three-step procedure including N-methylation, reduction of phosphine oxide, and Grignard addition to ester. Subsequently, **42** was applied as a bifunctional Lewis base organocatalyst in the formal [4 + 2] cyclization reaction between alkene **43** and 2,2′-dienone **44**. The corresponding spirooxindole **45** was obtained in >19:1 dr, 63% yield, and 89% ee.

#### Dynamic kinetic resolution of configurationally labile bridged biaryls

The catalytic asymmetric dynamic kinetic resolution (DKR) of configurationally labile bridged biaryls, pioneered by Bringmann and co-workers [45], has proven to be a powerful approach for synthesizing axially chiral biaryls, particularly in the challenging case of sterically hindered tetra-*ortho*-substituted scaffolds [46–47]. In this context, α-acidic isocyanides have been successfully employed as carbon nucleophiles in the DKR of various bridged biaryls bearing different linkages to give diverse nitrogen heterocycle-substituted atropoisomeric biaryls.

**Scheme 8:**
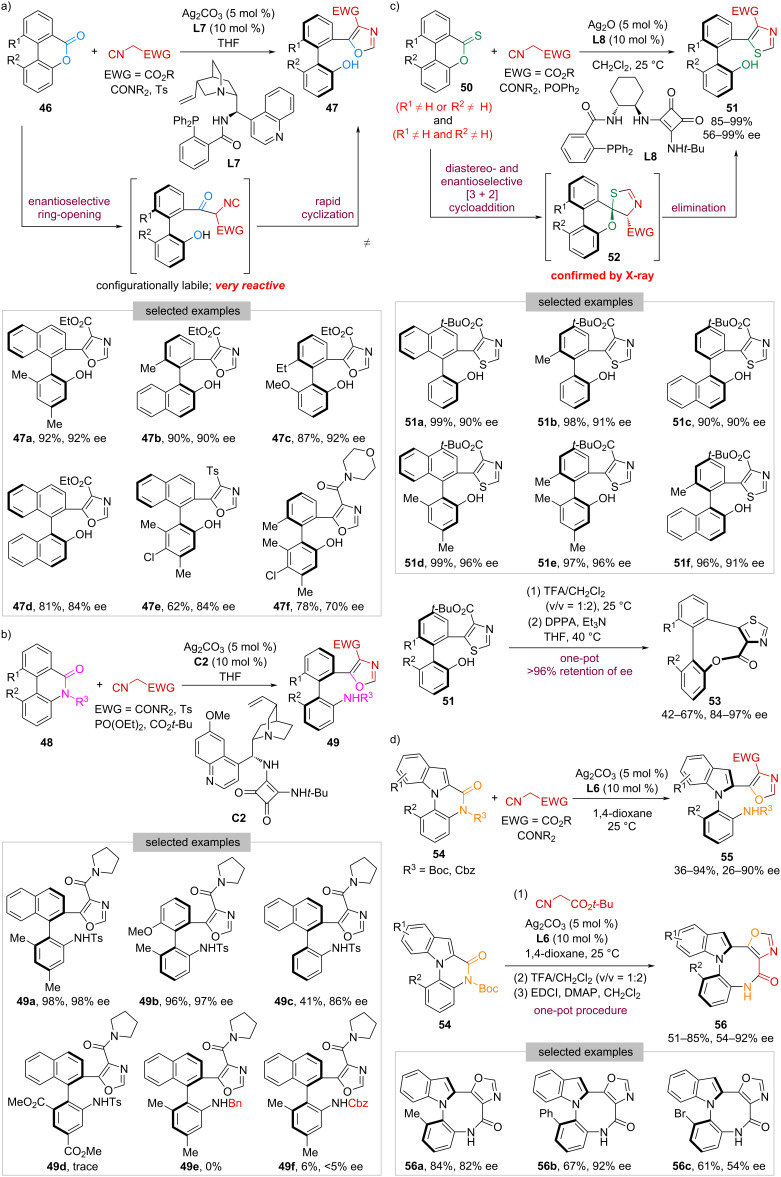
Dynamic kinetic resolution of bridged biaryls with α-acidic isocyanides.

In 2021, our group achieved the catalytic enantioselective DKR of biaryl lactones **46** with α-acidic isocyanides (Scheme 8a) [48]. By using Ag_2_CO_3_ and cinchonine-derived amino-phosphine **L7** as the catalyst, a wide range of oxazole-containing tetra-*ortho*-substituted axially chiral phenols **47** bearing diverse scaffolds, including naphthyl-phenyl (e.g., **47a**), phenyl-naphthyl (e.g., **47b**), biphenyl (e.g., **47c**), and binaphthyl (e.g., **47d**), were obtained in high yields with high enantioselectivities. In terms of isocyanides, isocyanoacetates with different ester groups, *p*-toluenesulfonylmethyl isocyanide (**47e**), and isocyanoacetamide (**47f**), were all compatible. It is noteworthy that this work represents the first example of catalytic asymmetric DKR of Bringmann’s lactones with carbon nucleophiles. The success lies in the tandem enantioselective ring-opening of lactones with α-acidic isocyanides, followed by a rapid cyclization driven by aromatization, overcoming the long-standing stereochemical leakage problem caused by the undesired lactol formation [45].

Encouraged by the above results, we turned our attention to biaryl lactams [49]. However, in this case, the inherent resonance stability of the amide bond makes the ring-opening process rather challenging. To solve this problem, we envisioned that a cooperative catalytic system merging silver catalysis and organocatalysis could be employed to activate both reactants simultaneously. After extensive screening, the combination of Ag_2_CO_3_ and quinidine-derived squaramide **C2** was identified to be the optimal choice of catalyst. A variety of *ortho*,*ortho*-disubstituted biaryl lactams **48** were facilely transformed into the corresponding tetra-*ortho*-substituted atropisomeric anilines **49** in high yields with excellent enantioselectivities (Scheme 8b). In contrast, lactams possessing only one *ortho* substituent suffered from much lower reactivity (e.g., **49c**), presumably due to the lack of sufficient torsional strain [50], whereas substrates bearing strong electron-withdrawing groups resulted in almost no reactivity (e.g., **49d**).

Additionally, it was found that the N-substituent R^3^ in **48** has a significant effect on both reactivity and enantioselectivity. While replacing Ts with Bn led to no reaction at all (**49e** versus **49a**), substituting it with Cbz gave the desired product **49f** in only 6% yield with <5% ee.

In 2022, we reported the discovery and development of a torsional strain-independent reaction between biaryl thionolactones **50** and α-acidic isocyanides (Scheme 8c) [51]. Using Ag_2_O and 1,2-diaminocyclohexane-derived phosphine-squaramide bifunctional ligand **L8** as the catalyst, a universal synthesis of tri- and tetra-*ortho*-substituted biaryl phenols **51** containing a thiazole moiety was achieved in 85–99% yield with 56–99% ee. It is worth mentioning that this work represents the first example of catalytic asymmetric DKR of biaryl thionolactones, getting rid of the pre-formation of stoichiometric Ru-complexes [52–53]. Mechanistic investigations indicated that this transformation proceeds through a two-step sequence promoted by the same catalyst: 1) the diastereo- and enantioselective [3 + 2] cycloaddition to generate spiro-*S*,*O*-ketal **52** with both axial and central chirality, followed by 2) ring-strain and aromatization-driven elimination, which elucidating the observed unusual torsional strain-independent reactivity. In addition, products bearing a *tert*-butyl ester group were smoothly converted into structurally novel axially chiral eight-membered lactones **53** in 42–67% yield with excellent retention of enantiopurity via an overall lactonization process.

In addition to C–C axial chirality, we have demonstrated that our Ag-catalyzed DKR protocol could be applied for the generation of C–N atropisomers by using *N*-arylindole lactams **54** as the cross-partner [54]. By employing Ag_2_CO_3_ and **L6** as the catalyst, axially chiral *N*-arylindoles **55** were synthesized in 36–94% yield with 26–90% ee (Scheme 8d). Building on such results, a one-pot procedure involving DKR, hydrolysis, and lactamization was developed, enabling a practical synthesis of structurally novel atropisomeric *N*-arylindoles **56** bearing an eight-membered lactam in 51–85% yield with 54–92% ee. Of note, these scaffolds exhibited remarkably large Stokes shifts, showing great potential in the development of fluorescent dyes.

#### Desymmetrization of prochiral compounds

Beyond DKR of bridged biaryls, our group has successfully applied α-acidic isocyanides in the catalytic asymmetric desymmetrization of substrates featuring a prochiral axis, realizing the preparation of structurally complex scaffolds possessing both axial and central chirality. Notably, in these cases, both issues of diastereo- and enantioselectivity are required to be addressed appropriately to prevent forming a complex mixture of stereoisomers.

**Scheme 9:**
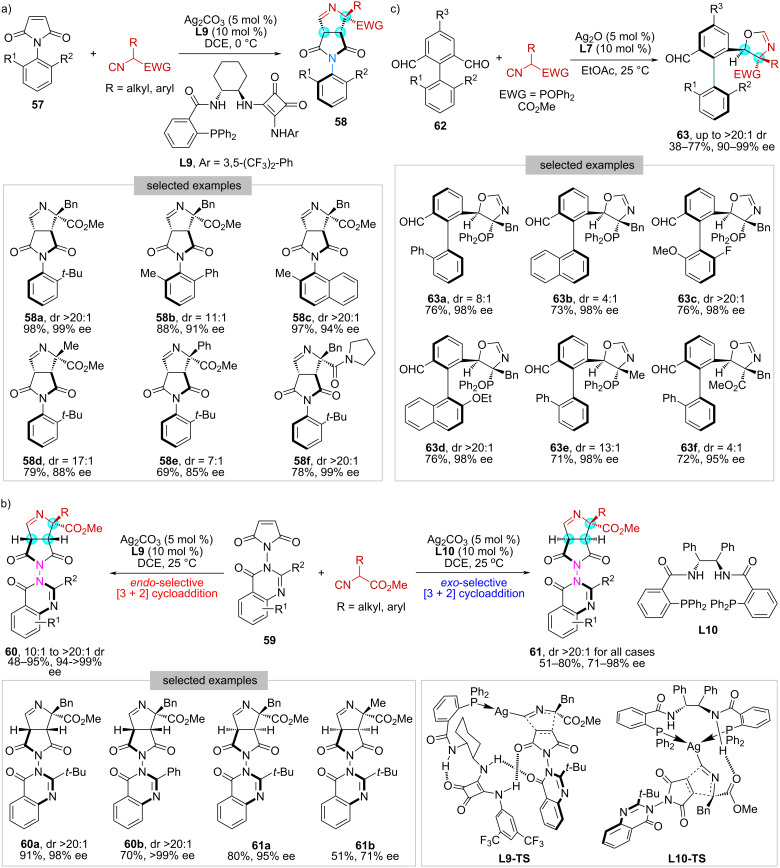
Desymmetrization of prochiral compounds with α-acidic isocyanides.

In 2022, our group developed a silver-catalyzed desymmetric [3 + 2] cycloaddition between prochiral *N*-aryl maleimides **57** and α-substituted α-acidic isocyanides (Scheme 9a) [55]. With Ag_2_CO_3_ and **L9** as the chiral catalyst, this reaction proceeded smoothly to produce highly functionalized bicyclic 1-pyrrolines **58** bearing a remote C–N stereogenic axis and three contiguous stereogenic carbon centers in high yields (up to 97%) with high stereoselectivities (up to >20:1 dr, 99% ee).

Subsequently, we expanded this methodology to prochiral *N*-quinazolinone maleimides **59**, achieving the simultaneous generation of N–N axial and central chirality within a single step (Scheme 9b) [56]. Of particular note, an interesting ligand-induced diastereodivergent profile was identified. While the employment of **L9** afforded *endo*-selective [3 + 2] cycloadducts **60**, using Trost ligand **L10** resulted in a complete reversal to the *exo*-cycloadducts **61**. DFT calculations were performed and indicated that these two ligands act in different ways in the cyclization process, providing explanations for such a diastereodivergence. Specifically, **L9** functions as a monodentate ligand, and the stronger ligand–substrate hydrogen-bonding interaction and smaller distortion of the ligand resulted in *endo-*cycloadducts (**L9-TS**). In contrast, Trost ligand **L10** serves as a bidentate ligand, and the smaller distortion of isocyanoacetate–Ag coordination combing with better Ag–C σ-orbital overlap led to *exo*-cycloadducts (**L10-TS**).

Except for constructing C–N and N–N axial chirality, we have developed a highly diastereo- and enantioselective method to access tri- and tetra-*ortho*-substituted biaryl aldehydes **63** bearing both C–C axial and central chirality (Scheme 9c) [57]. Key to this work relies on the implementation of an efficient Ag_2_O/**L7**-catalyzed desymmetric [3 + 2] cycloaddition of prochiral biaryl dialdehydes **62** with α-substituted α-acidic isocyanides. We have also demonstrated that the retained aldehyde functionality in **63** allowed for versatile derivatizations, such as reduction, reductive amination, condensation, and olefination, which further expanded the structural diversity of the resulting products.

## Summary and Outlook

The past few years have witnessed exciting progress in developing catalytic asymmetric transformations of isocyanides for generating architectures bearing axial, planar, helical, and inherent chirality. These advances not only offer efficient routes to enantioenriched non-central chiral compounds but also significantly broaden the utility of isocyanides in organic synthesis. Nevertheless, despite these notable accomplishments, this research field remains in its nascent stage with ample room for further exploration. First, existing studies have predominantly focused on the construction of axial chirality, while synthetic methods for other forms of non-central chirality, such as planar, helical, and inherent chirality, remain largely underdeveloped. To date, only a single example has been reported for each case, all of which are restricted to palladium-catalyzed isocyanide insertion reactions. Second, the catalytic systems employed thus far are relatively limited. Although transition metal catalysis, particularly with palladium and silver, has proven to be highly effective, expanding the scope as well as the activation mode of chiral catalysts could greatly enrich reaction types and accessible structural diversity. Third, further investigation into the potential applications of the resulting chiral products, e.g., biological activities and utility as chiral organocatalysts or ligands, warrants greater attention. We anticipate that considerable efforts in these directions would be crucial for advancing this field and fully unlocking the synthetic potential of isocyanides in the preparation of non-central chiral compounds.

## Data Availability

Data sharing is not applicable as no new data was generated or analyzed in this study.

## References

[1] Wencel-Delord J, Panossian A, Leroux F R, Colobert F (2015). Chem Soc Rev.

[2] Cheng J K, Xiang S-H, Li S, Ye L, Tan B (2021). Chem Rev.

[3] Carmona J A, Rodríguez-Franco C, Fernández R, Hornillos V, Lassaletta J M (2021). Chem Soc Rev.

[4] Schmidt T A, Hutskalova V, Sparr C (2024). Nat Rev Chem.

[5] López R, Palomo C (2022). Angew Chem, Int Ed.

[6] Laws D, Poff C D, Heyboer E M, Blakey S B (2023). Chem Soc Rev.

[7] Yang G, Wang J (2024). Angew Chem, Int Ed.

[8] Tanaka K, Morita F (2022). J Synth Org Chem, Jpn.

[9] Wang Y, Wu Z-G, Shi F (2022). Chem Catal.

[10] Huang Q, Tang Y-P, Zhang C-G, Wang Z, Dai L (2024). ACS Catal.

[11] Tang M, Yang X (2023). Eur J Org Chem.

[12] Luo Y, Luo S, Zhu Q (2025). J Org Chem.

[13] Dömling A, Ugi I (2000). Angew Chem, Int Ed.

[14] Dömling A (2006). Chem Rev.

[15] Sadjadi S, Heravi M M, Nazari N (2016). RSC Adv.

[16] Qiu G, Ding Q, Wu J (2013). Chem Soc Rev.

[17] Song B, Xu B (2017). Chem Soc Rev.

[18] Collet J W, Roose T R, Ruijter E, Maes B U W, Orru R V A (2020). Angew Chem, Int Ed.

[19] Kaur T, Wadhwa P, Bagchi S, Sharma A (2016). Chem Commun.

[20] Doraghi F, Baghershahi P, Gilaninezhad F, Darban N M Z, Dastyafteh N, Noori M, Mahdavi M (2025). Adv Synth Catal.

[21] Gulevich A V, Zhdanko A G, Orru R V A, Nenajdenko V G (2010). Chem Rev.

[22] Boyarskiy V P, Bokach N A, Luzyanin K V, Kukushkin V Y (2015). Chem Rev.

[23] Giustiniano M, Basso A, Mercalli V, Massarotti A, Novellino E, Tron G C, Zhu J (2017). Chem Soc Rev.

[24] van Berkel S S, Bögels B G M, Wijdeven M A, Westermann B, Rutjes F P J T (2012). Eur J Org Chem.

[25] Wang Q, Wang D-X, Wang M-X, Zhu J (2018). Acc Chem Res.

[26] Luo J, Chen G-S, Chen S-J, Li Z-D, Liu Y-L (2021). Chem – Eur J.

[27] Luo S, Xiong Z, Lu Y, Zhu Q (2018). Org Lett.

[28] Teng F, Yu T, Peng Y, Hu W, Hu H, He Y, Luo S, Zhu Q (2021). J Am Chem Soc.

[29] Wang X, Xu J, Luo Y, Wang Y, Huang J, Zhu Q, Luo S (2025). ACS Catal.

[30] Luo Y, Cheng S, Peng Y, Wang X, Li J, Gan C, Luo S, Zhu Q (2022). CCS Chem.

[31] Yu T, Li Z-Q, Li J, Cheng S, Xu J, Huang J, Zhong Y-W, Luo S, Zhu Q (2022). ACS Catal.

[32] Groebke K, Weber L, Mehlin F (1998). Synlett.

[33] Blackburn C, Guan B, Fleming P, Shiosaki K, Tsai S (1998). Tetrahedron Lett.

[34] Bienaymé H, Bouzid K (1998). Angew Chem, Int Ed.

[35] Hong S, Liu W, Zhang C, Yang X (2024). Sci Adv.

[36] Kamijo S, Kanazawa C, Yamamoto Y (2005). J Am Chem Soc.

[37] Larionov O V, de Meijere A (2005). Angew Chem, Int Ed.

[38] Zheng S-C, Wang Q, Zhu J (2019). Angew Chem, Int Ed.

[39] Zhang X, Liu Y-Z, Shao H, Ma X (2022). Molecules.

[40] Sun H-R, Sharif A, Chen J, Zhou L (2023). Chem – Eur J.

[41] He X-L, Zhao H-R, Song X, Jiang B, Du W, Chen Y-C (2019). ACS Catal.

[42] Barton D H R, Zard S Z (1985). J Chem Soc, Chem Commun.

[43] Yang H, Chen J, Zhou L (2020). Chem – Asian J.

[44] Min X-L, Zhang X-L, Shen R, Zhang Q, He Y (2022). Org Chem Front.

[45] Bringmann G, Breuning M, Tasler S (1999). Synthesis.

[46] Wang G, Huang J, Zhang J, Fu Z (2022). Org Chem Front.

[47] Wu C, Jin Y, Zhang X, Gao R, Dou X (2024). Eur J Org Chem.

[48] Qian L, Tao L-F, Wang W-T, Jameel E, Luo Z-H, Zhang T, Zhao Y, Liao J-Y (2021). Org Lett.

[49] Wang W-T, Zhang S, Tao L-F, Pan Z-Q, Qian L, Liao J-Y (2022). Chem Commun.

[50] Zhao K, Duan L, Xu S, Jiang J, Fu Y, Gu Z (2018). Chem.

[51] Luo Z-H, Wang W-T, Tang T-Y, Zhang S, Huang F, Hu D, Tao L-F, Qian L, Liao J-Y (2022). Angew Chem, Int Ed.

[52] Schenk W A, Kümmel J, Reuther I, Burzlaff N, Wuzik A, Schupp O, Bringmann G (1999). Eur J Inorg Chem.

[53] Bringmann G, Wuzik A, Kümmel J, Schenk W A (2001). Organometallics.

[54] Tao L-F, Huang F, Zhao X, Qian L, Liao J-Y (2023). Cell Rep Phys Sci.

[55] Zhang S, Luo Z-H, Wang W-T, Qian L, Liao J-Y (2022). Org Lett.

[56] Wang W-T, Zhang S, Lin W, Luo Z-H, Hu D, Huang F, Bai R, Lan Y, Qian L, Liao J-Y (2024). Org Chem Front.

[57] Huang F, Tao L-F, Liu J, Qian L, Liao J-Y (2023). Chem Commun.

